# The Evaluation of Dermatological Disease Profiles of Neurosurgery Inpatients: A Tertiary Clinic Experience

**DOI:** 10.7759/cureus.56633

**Published:** 2024-03-21

**Authors:** Berkay Temel, Ozge Orenay, Nermin Karaosmanoglu, Ayhan Tekiner

**Affiliations:** 1 Department of Dermatology, Health Sciences University, Ankara Training and Research Hospital, Ankara, TUR; 2 Department of Neurologic Surgery, Health Sciences University, Ankara Training and Research Hospital, Ankara, TUR

**Keywords:** nitrofurazone, contact dermatitis, seborrheic, neurosurgery, dermatology

## Abstract

Background: Both the skin and neuronal systems originate from the ectoderm. In patients hospitalized for neurosurgery, their skin may be affected by genetic and environmental factors.

Objective: This study researched disease relationships by evaluating the profile of hospitalized neurosurgery patients who consulted with dermatology in a tertiary clinic (Neurosurgery Clinic, Ankara Training and Research Hospital, Ankara).

Methods: This study included hospitalized neurosurgery patients who consulted with dermatology. Age, gender, type of hospitalization, neurosurgical diseases, and dermatology diseases were selected as study variables. The medical health records of the patients were retrospectively scanned and analyzed.

Results: A total of 172 consultations were analyzed. The mean age of patients was 44.7 years old ranging from 1 year to 99 years old. The percentage of male patients was 54.7%; 25.5% of the patients were hospitalized for cerebral vascular diseases, 30.2% for spinal diseases, 22.1% for tumors, 12.2% for infections, and 1.2% for other neurosurgical diseases. The most commonly diagnosed dermatological disease in patients was drug eruptions (18.6%), followed by seborrheic dermatitis (16.2%) and contact dermatitis (14.5%).

Conclusion: According to this study, the most commonly diagnosed dermatological diseases in neurosurgery inpatients were drug eruption, contact dermatitis, and seborrheic dermatitis. The results of this study may be helpful in terms of neurosurgical training planning and treatment management.

## Introduction

Dermatology includes a wide range of diseases. While some of them are essentially skin disorders, others are manifestations of systemic diseases. Dermatology consultation is sometimes required in patients who are hospitalized for non-dermatological disorders. Although some patients may have dermatological disorders prior to admission to the hospital, others may develop them during or after hospitalization. These dermatological problems can sometimes interfere with the treatment of conditions that require hospitalization. It is hard to solve this condition in a non-dermatologic department without a dermatology consultation. As a result, dermatological consulting is essential [1].

The embryonic development of the skin and central/peripheral nerve systems comes from the ectoderm. Some diseases may impact both systems at once. Several skin diseases might present with neurological symptoms such as stroke, vascular malformations, malignancies, and epilepsy at the same time [2]. Some dermatological conditions occur due to factors in the environment (such as drug eruptions) and may affect the management of neurological disease [3]. It's critical to identify or suspect dermatological disease in order to reach the best diagnosis and initiate the most effective method of treatment. As a result, dermatology consultations are crucial for clinics that manage neurological diseases like neurosurgery.

To date, there have been limited studies evaluating the profile of neurosurgery patients consulted with dermatology [3-6]. In this study, the aim was to research disease relationships by evaluating the profile of hospitalized neurosurgery patients consulted with dermatology in a tertiary hospital (Neurosurgery Clinic, Ankara Training and Research Hospital, Ankara).

## Materials and methods

This study included hospitalized neurosurgery patients who consulted with dermatology between August 2013 and August 2023. The study was approved by the Ankara Training and Research Hospital Ethics Committee and was in accordance with the Declaration of Helsinki. (E-23-1393).

Patient selection and inclusion criteria

The design of the study was a retrospective cohort. The medical health records of the patients were scanned. Patients were planned to be included in the study according to the inclusion criteria. The inclusion criteria of the study were that medical records were accessible and complete in terms of study variables. The exclusion criterion of the study was missing information in terms of study variables.

Study variables

Age, gender, type of hospitalization, neurosurgical diseases, and dermatology diseases were selected as study variables. The age was grouped into three categories. There were under 18-year-olds, 18-65-year olds, and over 65-year-olds. The type of hospitalization was divided into two categories. These were inpatient and intensive care units. The neurosurgical diseases were divided into five categories. These were cerebral vascular diseases, spinal diseases, tumors, infections, and others. The dermatological diseases were divided into four categories. These categories were dermatitis, infection, inflammatory, and others. These categories were created using ICD-10 codes.

Study plan

The patient's electronic medical records were examined retrospectively by two authors. Study variables were recorded. The analysis was performed using appropriate statistical methods.

Statistical analysis

Research data was analyzed using IBM SPSS Statistics for Windows, Version 22 (Released 2013; IBM Corp., Armonk, New York, United States). Descriptive data was saved as mean, frequency distribution, and percentile. 

## Results

Main characteristics of the study

The mean age of patients was 44.7 years. The 0-18 age group had 10.5% (n=18) of patients. The 18-65 age group had 63.9% (n=110) of patients. The over-65 age group had 25.6% (n=44) of patients. The ratio of male patients was 54.7% (n=94). Sixty-one percent (n=105) of the patients were consulted from the inpatient units and 39% (n=67) from the intensive care units; 25.5% (n=44) of the patients were hospitalized for cerebral vascular disease, 30.2% (n=52) for spinal disease, 22.1% (n=38) for tumors, 12.2% (n=21) for infections, and 1.2% (n=17) for other neurosurgical diseases. The list of subgroup diseases is shown in Table 1. Other spinal diseases were tethered cord (n=3, 1.7%), spinal stenosis (n=9, 5.2%), and spondylolisthesis (n=3, 1.7%). Other neurosurgical diseases were hydrocephalus (n=15, 8.7%) and dermoid cyst (n=2, 1.1%).

**Table 1:** The main characteristics of the study SD: Standard deviation The data has been represented as n(%) and mean±SD

	Patients (n=172)
Age, mean±SD year	44.7 ±13,2
Age groups, n(%)	
0-18 years	18 (10.5)
18-65 years	110 (63.9)
>65 years	44 (25.6)
Gender, n(%)	
Male	94 (54.7)
Female	78 (45.3)
Type of hospitalization n(%)	
Inpatient unit	105 (61)
Intensive care unit	67 (39)
Neurosurgical diseases, n(%)	
Cerebral vascular diseases, n(%)	44 (25.5)
Intracranial hemorrhagic disorders	36 (20.9)
Cerebral infarction or transient ischemic attack	1 (0.6)
Cerebral aneurysm	7 (4.1)
Spinal diseases, n(%)	52 (30.2)
Herniation	16 (9.3)
Spinal fracture and trauma	21 (12.2)
Others	15 (8.7)
Tumor, n(%)	38 (22.1)
Infectious disease, n(%)	21 (12.2)
Others, n(%)	17 (9.8)

Dermatological findings and disease profile of patients

**Figure 1:**
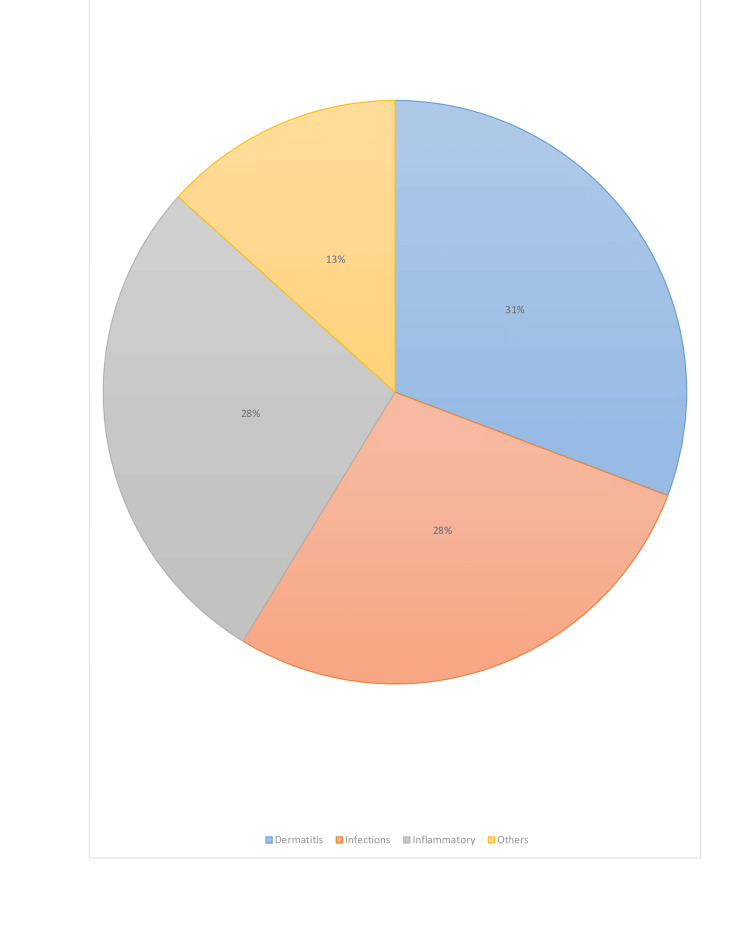
The distribution of dermatologic diseases

The most seen dermatological finding in 32.6% (n=56) of the patients was macules/patches, followed by papules (n=36, 20.9%) and plaques (n=47, 27.3%). The most commonly diagnosed dermatological disease in patients was drug eruption (n=32, 18.6%), followed by seborrheic dermatitis (SD) (n=28, 16.2%) and contact dermatitis (n=25, 14.5%). Drug eruptions were caused by cefazolin, piperacillin/tazobactam, diclofenac, naproxen, ceftriaxone, and levetiracetam. The most common form of drug eruption was morbilliform (100%). Contact dermatitis was caused by topical nitrofurazone (n=12, 48%), topical diclofenac (n=4, 16%), plaster tape (n=4, 16%), and diapers (n=5, 20%). Twenty percent (n=5) of the contact dermatitis patients had irritant contact dermatitis, while 80% (n=20) had allergic contact dermatitis. The rest of the dermatological findings and disease list are shown in Table 2 and Figure 1.

**Table 2:** Dermatologic disease profiles of study participants The data has been represented as n(%)

Dermatological findings, n(%)	
Macules/Patches	56 (32.6)
Papules	36 (20.9)
Plaques	47 (27.3)
Bullas	5 (2.9)
Vesicule and pustules	11 (6.4)
Petechia/purpura	5 (2.9)
Ulcer	11 (6.4)
Dermatologic diseases, n(%)	
Dermatitis	53 (30.7)
Contact dermatitis	25 (14.5)
Seborrheic dermatitis	28 (16.2)
Infections	48 (27.9)
Cutaneous bacterial diseases	9 (5.2)
Cutaneous viral diseases	12 (7)
Cutaneous fungal diseases	23 (13.4)
Scabies	4 (2.3)
Inflammatory	48 (27.9)
Drug eruption	32 (18.6)
Urticaria	2 (1.2)
Papulosquamous diseases	3 (1.7)
Bullous diseases	1 (0.6)
Alopecia areata	1 (0.6)
Folliculitis	9 (5.2)
Others	23 (13.3)
Hemangioma	1 (0.6)
Genetic skin diseases	1 (0.6)
Nevus	4 (2.3)
Cutaneous ulcers	17 (9.9)

The most diagnosed three dermatological diseases in neurosurgery inpatients

Contact dermatitis is the most commonly diagnosed dermatological disease in patients with intracranial hemorrhagic diseases, cerebral aneurysms, tumors, other spinal diseases, and other neurosurgical patients. SD is the most commonly diagnosed dermatological disease in patients with cerebral infarcts. Drug eruption was the most commonly diagnosed dermatological disease diagnosed in patients with herniations, spinal fractures/trauma, and infections. The list of diagnosed three dermatological diseases in patients is shown in Table 3.

**Table 3:** The most seen three dermatological diseases in neurosurgery inpatients

Neurosurgical diseases	Dermatological diseases
Cerebral vascular diseases	
Intracranial hemorrhagic disorders	1-Contact dermatitis
	2- Cutaneous ulcers
	3- Seborrheic dermatitis
Cerebral infarction or transient ischemic attack	1-Seborrheic dermatitis
Cerebral aneurysm	1-Contact dermatitis
	2- Seborrheic dermatitis
	3- Cutaneous bacterial diseases
Spinal diseases	
Herniation	1-Drug eruption
	2-Contact dermatitis
	3-Seborrheic dermatitis
Spinal fracture and trauma	1-Drug eruption
	2-Seborrheic dermatitis
	3-Contact dermatitis
Others	1-Contact dermatitis
	2- Cutaneous fungal diseases
	3- Seborrheic dermatitis
Tumor	1-Contact dermatitis
	2- Cutaneous fungal diseases
	3- Seborrheic dermatitis
Infectious disease	1-Drug eruption
	2-Seborrheic dermatitis
	3-Contact dermatitis
Others	1-Contact dermatitis
	2- Seborrheic dermatitis
	3- Cutaneous bacterial diseases

## Discussion

Dermatology currently provides primarily outpatient healthcare services. Although cosmetic dermatology has become more popular, clinical dermatology is still essential. Inpatient consultation is an essential part of clinical dermatology. Patients who are hospitalized for non-dermatological diseases can be consulted for dermatological diseases. Recognizing the consultation profile is important for appropriate patient management. There were a considerable number of studies examining dermatological consultations in the literature [3,5,7-11]. However, there was a lack of information and studies about dermatological diseases in neurosurgery inpatients [6]. This study aimed to contribute to the literature in this regard.

In the literature, the relationship between medical departments and dermatological diseases has been analyzed many times based on consultations. In these studies, internal medicine, pediatrics, infectious diseases, and emergency departments were the most frequently requested dermatological consultations. Dermatitis, skin infections, and drug eruption were the most commonly diagnosed dermatological diseases in these studies [3,5,7-11]. There was limited information about neurosurgery in these studies. A lower percentage of requests from neurosurgery has been documented in some of these studies [7,8,10]. It's interesting to note that neurosurgery was one of the most requested dermatology consultations according to the prior two studies [6,11]. In our study, consultations requested from neurosurgery constituted a small proportion of all consultations.

In a study with a similar purpose to ours, dermatological diseases of neurosurgery inpatients were examined. The mean age was 58.6 years with male dominance (51.8%). These results were consistent with ours. Cerebrovascular diseases were the most common reason for hospitalization, followed by spinal diseases and tumors in the previous study [6]. However, in our study, the most common reason for hospitalization was spinal disease. Additionally, the percentage of tumor patients was lower compared to our study. This may be due to the difference in the number of patients and the difference in physician profiles experienced in tumor surgery between the neurosurgery centers of study.

Eczematous dermatitis (not contact dermatitis or SD) was the most diagnosed dermatologic disease followed by cutaneous fungal infections and drug eruptions in the previous study. In our study, we found the same dermatological disease profile. However, the order was different [6]. We found that the most diagnosed dermatologic diseases were drug eruption, contact dermatitis, and SD.

Drug eruptions, also known as toxidermia, are cutaneous symptoms that occur as a result of systemic drug administration. These reactions range from mild erythematous skin lesions to far more serious reactions like toxic epidermal necrosis [12]. Morbilliform or erythematous maculopapular eruptions account for around 40% of all drug eruptions. Antibiotics (beta-lactams, sulfonamides), nonsteroidal anti-inflammatory medicines (NSAIDs), antiepileptics (carbamazepine, hydantoins), and allopurinol are the most commonly caused agents. The lesions typically form on the trunk or in areas of pressure or trauma first. They spread to the extremities, usually in a symmetrical pattern [13]. In our study, all patients with drug eruption had a morbilliform type. Additionally, the culprit drugs of our study (piperacillin tazobactam, naproxen, and diclofenac) were compatible with the literature. Drug eruptions were found in most patients with neurosurgical infectious diseases, herniation, and spinal fracture/ trauma. In one study, Kim et al. showed the same relationship between infectious diseases and drug eruption in neurosurgical inpatients [11]. This is logical because these patients are more likely to use antibiotics and NSAIDs. In the literature, some drugs induced drug eruption in neurosurgical patients in addition to our causal agents like temozolomide and cephalexin [14,15]. We suggest that neurosurgeons should investigate drug eruption and consult with dermatology in patients who have an erythematous maculopapular rash after taking antibiotics or NSAIDs.

Contact irritants and allergens are chemicals or metal ions that have negative impacts on the skin. Irritant contact dermatitis and allergic contact dermatitis are the two types of contact dermatitis [16,17]. In our study, the causative agents of contact dermatitis were found as topical nitrofurazone, topical diclofenac, plaster tape, and diapers. Nitrofurazone is a topical antibacterial agent having broad-spectrum action. In our country, surgeons, and family physicians choose it for chronic leg ulcers, superficial skin infections, ulcers, burns, and other types of chronic dermatitis. The ointment form of nitrofurazone comprises 0.2% nitrofurazone and the vehicles polyethylene glycol (PEG) 300, PEG 1000, and PEG 4000. Nitrofurazone sensitization is widely reported in the literature [18]. Because of the high incidence of allergic reactions, the use of nitrofurazone has been largely discontinued in Western countries [19]. Some agents induced contact dermatitis in neurosurgical patients in addition to our causal agents like the cervical collar and mastisol (liquid medical adhesive used to secure dressings and tapes) [20,21]. In our study, contact dermatitis was diagnosed mostly in patients with intracranial hemorrhagic disease, cerebral aneurysms, tumors, other spinal diseases, and other neurosurgical patients. The possibility of being bedridden for a long time and having to undergo many surgeries is more likely in these diseases. Based on this data, we suggest neurosurgeons avoid using unnecessary topical antibiotics and NSAIDs in wound care and use allergy-free diapers and faster tapes.

SD is a common inflammatory skin disease characterized by a papulosquamous morphology in sebaceous gland-rich areas, such as the scalp, face, and body folds. Scaling, erythema, and itching are common symptoms of SD [22]. The relationship between neurological/neurosurgical diseases and SD has been examined many times in the literature. Particularly, the relationship between Parkinson's disease and SD was emphasized [23]. Reed et al. reported a significant incidence of SD following spinal cord injury as a result of changed sebum secretion, dermatophytosis, and sweat secretion alterations. In addition, the deposition of sebum and scales on improperly cleaned skin caused by prolonged immobilization was proposed as a probable cause of SD in this study [24]. Kim et al. reported a relationship between intracranial hemorrhage and SD. It has been suggested that the quadriplegic condition seen in ICH patients could be one of the causes [6]. It is clear that more studies are needed to examine the relationship between SD and neurosurgical diseases.

There are some limitations in this study. This study was a retrospective study with a limited number of participants. Additionally, this study was single-center and there was no control group in the study. The strength of our study was that it is one of the few studies on this subject in the literature.

## Conclusions

According to this study, the most commonly diagnosed dermatological diseases in neurosurgery inpatients were drug eruption, contact dermatitis, and SD. The results of this study may be helpful in terms of neurosurgical training planning and treatment management. However, studies with more participants are needed to examine the relationship between dermatological and neurosurgical diseases.
